# Atypical Lindenane-Type Sesquiterpenes from *Lindera myrrha*

**DOI:** 10.3390/molecules25081830

**Published:** 2020-04-16

**Authors:** Thuc-Huy Duong, Mehdi A. Beniddir, Nguyen T. Trung, Cam-Tu D. Phan, Van Giau Vo, Van-Kieu Nguyen, Quynh-Loan Le, Hoang-Dung Nguyen, Pierre Le Pogam

**Affiliations:** 1 Department of Chemistry, University of Education, 280 An Duong Vuong Street, District 5, Ho Chi Minh City 700000, Vietnam; huydt@hcmue.edu.vn; 2 Équipe “Pharmacognosie-Chimie des Substances Naturelles”, BioCIS, Université Paris-Sud, CNRS, Université Paris-Saclay, 5 Rue J.-B. Clément, 92290 Châtenay-Malabry, France; mehdi.beniddir@universite-paris-saclay.fr; 3 Laboratory of Computational Chemistry and Modelling (LCCM), Quy Nhon University, Quy Nhon City 55100, Vietnam; nguyentientrung@qnu.edu.vn (N.T.T.); phandangcamtu@qnu.edu.vn (C.-T.D.P.); 4 Bionanotechnology Research Group, Ton Duc Thang University, Ho Chi Minh City 700000, Vietnam; vovangiau@tdtu.edu.vn; 5 Faculty of Pharmacy, Ton Duc Thang University, Ho Chi Minh City 700000, Vietnam; 6 Institute of Fundamental and Applied Sciences, Duy Tan University, Ho Chi Minh City 700000, Vietnam; nguyenvankieu2@duytan.edu.vn; 7 Faculty of Natural Sciences, Duy Tan University, Da Nang 550000, Vietnam; 8 Institute of Tropical Biology, Vietnam Academy of Science and Technology, Ho Chi Minh City 700000, Vietnam; lqloan@itb.ac.vn; 9 NTT-High tech Institute, Nguyen Tat Thanh University, Ho Chi Minh City 700000, Vietnam

**Keywords:** *Lindera*, sesquiterpene, lindenane, DFT-NMR

## Abstract

Two new lindenane sesquiterpenes were obtained from the roots of *Lindera myrrha*. These compounds were structurally elucidated by HRMS data, extensive NMR analyses, and comparison between experimental and theoretical ^13^C-NMR data. Myrrhalindenane A is the first monomeric seco-d lindenane displaying a non-rearranged, cyclohexanic C-ring. Myrrhalindenane B is the second occurrence of an angular lindenane-sesquiterpene related to a C_6_-C_7_ lactonization.

## 1. Introduction

*Lindera* is a core genus of the Litseeae tribe of the Lauraceae family [1]. Many *Lindera* plants are of salient economical interest for soap and lubricant manufacturing (especially *Lindera communis* and *Lindera glauca*) owing to their elevated fatty oil content, while others are used to produce fragrances, species, and even building timber. As to ethnopharmacological claims, *Lindera aggregata* is included in various preparations of the Chinese Pharmacopoeia for treating urinary system diseases and inflammatory-related health hazards [2]. Other plants are also used in folk medicine such as *Lindera umbellate*, which is endowed with antispasmodic properties and has beneficial effects on gastric ulcers, cholera, and beriberi [3]. Fueled by the diverse interests lying in these plants, a wealth of skeletons were reported to have occurred in this well-studied family, the most represented of which include sesquiterpenes (mainly lindenanes, eudesmanes, and germacranes), and aporphine alkaloids, along with some typical α-methylene-γ-butyrolactones collectively known as butanolides, and a few emblematic polysubstituted cyclopentanediones designated as lucidones [2]. Within this thoroughly studied genus, *Lindera myrrha* (Lour.) Merr., a small shrub common in central Vietnam, long remained unstudied. Conducted in 1994, the first phytochemical investigation dedicated to this species led to the isolation of a suite of aporphine alkaloids, including a new noraporphine, oduocine; and a new oxaporphine, oxoduocine [4]. A novel dihydroisocoumarin, lindermyrrhin, was further described from *L. myrrha* [5], but as far as can be ascertained, its terpene content remained unstudied. With this in mind, our study focused on the sesquiterpenes of *L. myrrha* roots, leading to the isolation of two new structures: myrrhalindenanes A and B. The structures of the isolated compounds **1** and **2** were elucidated by the interpretation of their spectroscopic data and by comparison with those described in the literature.

## 2. Results and Discussion

Compounds **1** and **2** were isolated from the methanol extract of *L. myrrha* by repeated chromatographic fractionations, including column chromatography, size-exclusive column chromatography, and preparative TLC.

**Figure 1:**
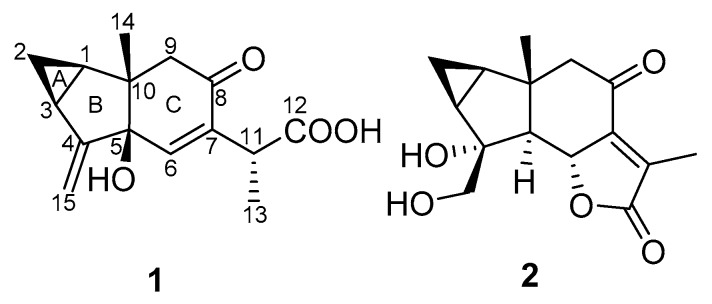
Chemical structures of compounds **1** and **2**.

**Figure 2:**
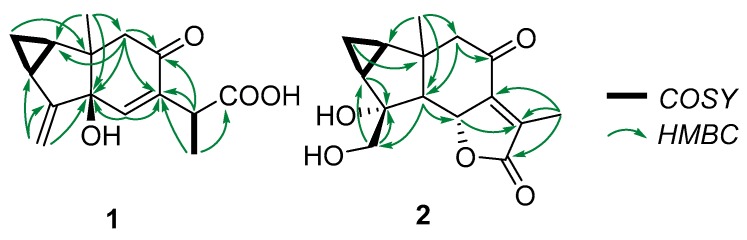
COSY and key HMBC correlations of compounds **1** and **2**.

**Figure 3:**
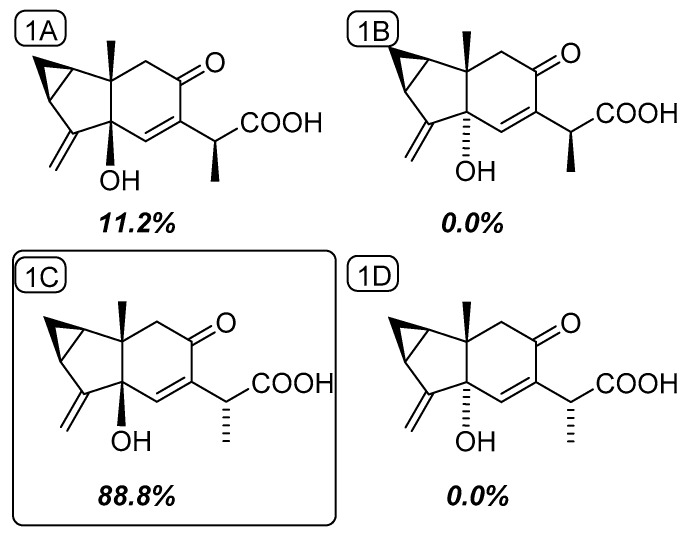
Chemical structures of the four possible diastereoisomers of compound **1** along with their respective DP4 probabilities.

Compound **1** was isolated as a white, amorphous solid. Its molecular formula was determined to be C_15_H_18_O_4_ from its HRESIMS ion at *m*/*z* 285.1090 [M + Na]^+^ (calculated for C_15_H_18_O_4_Na, 285.1097). The ^13^C-NMR spectrum, along with HSQC data, exhibited 15 signals for carbons consisting of one carbonyl, one carboxyl, two olefinic quaternaries, an oxygenated tertiary carbon, an olefinic methine, an exo-methylene, three methines, and a quaternary carbon (Table 1). These functionalities accounted for 4 indices of H deficiency, defining the tricyclic scaffold of **1** (Figure 1). The ^1^H-^1^H correlation spectroscopy spectrum of **1** showed a proton spin system of a 1,2-disubstituted cyclopropane ring (δ_H_ 1.46 (H-1); δ_H_ 0.71/1.52 (H_2_-2); and δ_H_ 2.00 (H-3)) (Supplementary Materials). These structural features were evocative of a lindenane-type sesquiterpene [6]. The cautious analysis of the 2D-NMR spectra revealed a polycyclic framework embedded with a sterically congested cyclopentane, as deduced from the HMBC correlations from the angular methyl group at δ_H_ 1.13 (H_3_-14) to the carbons resonating at δ_C_ 31.1 (C-1), δ_C_ 76.5 (C-5), and δ_C_ 51.2 (C-9), and from the exo-methylene that was located at C-4 based on long-range heteronuclear crosspeaks between the olefinic protons at δ_H_ 4.99/5.17 (H_2_-15) to C-3 (δ_C_ 23.4) and C-5 (δ_C_ 76.5). The chemical shift of the isolated diastereotopic methylene group at δ_H_ 2.31/2.39 (each 1H, d, *J* = 15.5 Hz) hinted at it being vicinal to a carbonyl function. This tentative assignment was supported by the HMBC crosspeak from H_2_-9 to the carbon resonating at δ_C_ 197.7 (C-8). Altogether, these spectroscopic data left no choice but to introduce a Δ^6,7^ moiety. The C-6 location of the olefinic proton was validated based on the HMBC correlations from H-6 to C-5, the quaternary olefinic carbon resonating at δ_C_ 163.1 (C-7), and to C-8. In the end, the C-9 location of the side chain was established owing to the HMBC correlations from the methine at δ_H_ 3.50 (H-11) to both C-7 and C-8. This methine was deduced to have been substituted by a methyl and a carboxylic acid group based on (i) the COSY crosspeak between this and the methyl protons at δ_H_ 1.13 (CH_3_-14), and (ii) the HMBC correlation from these methyl protons to both C-7 and the carbon resonating at δ_C_ 174.0 (C-12). These spectroscopic features determined the planar structure of **1**, namely myrrhalindenane A, as indicated in Figure 2. The NOESY correlations between H_2_-2 and H_3_-14 determined their synfacial orientation. Aside from the doubts regarding C-5 configuration, the absolute configuration assignment of C-11 represented a vexing problem in its achiral environment. These spectroscopic features led us to consider four different stereochemical arrangements, as indicated in Figure 3. DFT-NMR chemical shift calculations and the subsequent DP4 probability method [7] were performed on these different candidates. This DP4 application demonstrated the structural equivalence of **1** with diastereoisomer 1C with 88.8% probability (Figure 3).

**Figure 4:**
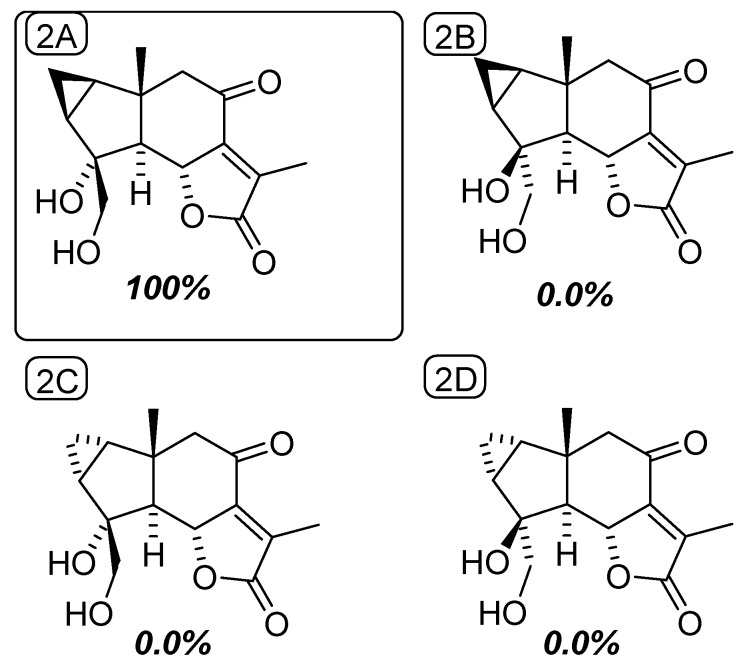
Chemical structures of the four possible diastereoisomers of compound 2 along with their respective DP4 probabilities.

Compound **2** was obtained as a white, amorphous solid. Its molecular formula, C_15_H_18_O_5_, was established from the sodiated ion peak at *m/z* 301.1047 (calculated for C_15_H_18_O_5_Na), differing from compound **1** by an additional oxygen atom. Notwithstanding their common lindenane core, the NMR data revealed some salient structural differences between these compounds. The ^13^C-NMR data revealed the lack of the exo-methylene moiety and the loss of the olefinic proton although a tetrasubstituted double bond could be identified. In line with this latter point, the downfield ^1^H chemical shift of the signal related to the methyl CH_3_-13 (δ_H_ 1.92 vs. 1.26), combined with the shielding of the corresponding carbon (δ_C_ 9.8 vs. 16.6) were evocative of its location on a double bond [8]. Conversely, the ^1^H-NMR spectrum displayed further signals corresponding to a tertiary methine at δ_H_ 2.27 (1H, d, *J* = 12.5 Hz), coupled with an oxygenated methine at δ_H_ 5.03 (1H, d, *J* = 12.5 Hz). Likewise, an additional set of oxygenated diastereotopic methylene at δ_H_ 3.80/3.67 could be identified, as well as a new tertiary oxygenated methine at δ_C_ 80.1. Along with the unchanged carbonyl moieties at δ_C_ 197.7 and 173.9, these functionalities represented three indices of hydrogen deficiency, determining the tetracyclic appendage of **2**. The oxygenated methylene could be located at C-4 based on the long-range heteronuclear correlations from these protons to C-3 (δ_C_ 28.7), C-4 (δ_C_ 80.1), and C-5 (δ_C_ 63.8). The joint HMBC correlations from the methyl protons at δ_H_ 1.11 and of the diastereotopic methylene signals at δ_H_ 2.62/2.67 (each 1H, d, *J* = 16 Hz) to the carbon resonating at δ_C_ 63.8, validated the occurrence of a methine at this specific position (C-5). The chemical shift of C-4 (δ_C_ 80.1) defined the presence of a hydroxy group on it. Such B-ring structures are recurrent within lindenane sesquiterpenes, falling into the third subtype defined by Du [9]. The tetracyclic core of **2**, and the unchanged chemical shifts of both C-8 and C-9 left no possibility but to introduce an additional α-methyl-Δ^α,β^-γ lactone fused ring at C-6/C-7. This assumption was validated by the correlations from the olefinic-located methyl at δ_H_ 2.27 to the quaternary carbons C-7 (δ_C_ 154.6) and C-11 (δ_C_ 132.1), to the carbonyl-type carbon C-12 (δ_C_ 173.9), and from the oxymethine proton H-6 to C-11. These spectroscopic data were fully consistent with those of formerly reported sesquiterpene lactones [10,11]. The antiperiplanar orientation of H-5 and H-6 could be determined from the magnitude of the coupling constant value (*J* = 11.5 Hz) [11]. Having in mind, i) the consensual trans arrangement of the hydrindane system in lindenane sesquiterpenes, and ii) the antifacial orientations of H-5 and H-6, only left the configuration of C-4 pending assignment [12,13]. A preferred configuration for C-4 prevails with a β-OH group and an α-oxygenated methylene moiety, so that Du’s lindenane sesquiterpene subtypes define the absolute configuration of this stereocenter [9]. Nevertheless, exceptions were reported throughout literature [11,14,15], so assigning the configuration of these positions based solely on biosynthetic considerations is not a relevant approach to reliably establish the configuration of such compounds. To remedy this, DFT-NMR calculations and subsequent ^13^C-NMR data comparison of the two possible epimers against the experimental data set, resulted in the prediction of diastereoisomer 2A with 100% probability (Figure 4).

Compounds **1** and **2** were found to be unstable on storage. After three days at room temperature, both had undergone ca. 70% decomposition to yield a mixture of products. The minute amounts of compound precluded any further repurification attempt. In these conditions, the recorded ECD spectra provided no clear-cut match, irrespective of the absolute configuration used in TDDFT. This observation is in line with precedents having outlined the inherent instability of lindenane ring system [16,17], which was occasionally reported in the course of former phytochemical investigations [18]. Despite the lack of spectroscopic evidence, the consensual β-orientation of both the methyl and cyclopropyl functions not only in *Lindera* species [2], but also within the Chloranthaceae plants that produce a much higher number of these sesquiterpenes [19,20], gave strong support to the preferred absolute configuration depicted in Figure 1.

The determined A/B ring substitution pattern of myrrhalindenane A is common among lindenane sesquiterpenes, falling into the lindenane sesquiterpenoid subtype I, as defined by Du and co-workers [9]. Conversely, the occurrence of oxygenated substituents at C-5 is rather uncommon among lindenane sesquiterpenes, since this position is often substituted by an α-disposed hydrogen atom, or is unsaturated due to either a Δ^4,5^ or a Δ^5,6^ function [13]. A few structures were however reported to contain an oxygenated substituent at C-5 such as sarcandralactone A, which revealed a 5β-OH group [21] or the dimeric sarcandrolide F that exhibits a 5β-OOH group [22]. The side chain located at C-7 can be assumed to arise from the hydrolysis of a 2-methyl-2-butyrolactone or a 2-methylbutyrolactone D ring related to the canonical lindenane skeleton. Only a few seco-d lindenanes have been reported to date. Some such compounds were formerly described in the *Lindera* species as strychnilactone [23], lindenanolide G [24], and linderagalactones B and C [25]. Nevertheless, all these structures undergo later rearrangement to afford a α-pyrone C-ring, therefore differing from the currently reported carbon skeleton. These compounds also differ from **1** by the constant occurrence of a Δ^7,11^ moiety. Remarkably, a wealth of seco d-lindenanes were reported within lindenane sesquiterpenoid [4 + 2] dimers, especially from the *Sarcandra* species, e.g., sarcandrolides [22,26]; and various *Chloranthus* plant species such as shizukaol species [27,28,29], chlorahololides [30,31], spicachloranthins E and F [32], and chlorajaponilides [33], among many others. The biosynthesis of dimeric lindenane sesquiterpenes is deemed to proceed via a Diels–Alder reaction with Δ^4,15^ and Δ^5,6^ representing the diene reactive unit [34]. Furyldiene lindenanes and, more generally speaking, molecules displaying these structural features rendering them prone to undergoing Diels–Alder addition, seem to be too unstable to be isolable [9]. This inherent reactivity towards dimerization most likely accounts for **1** being the first reported seco d-lindenane monomer, which can be readily related to its Δ^6,7^ function that prevents it from dimerizing. Lindermyrrhin B (**2**) is the second example of a 3/5/6/5 tetracyclic lindenane-type sesquiterpene lactone formed at C-6 and C-7, with the first such occurrence being reported from *Xanthium sibericum* (Asteraceae) [11].

## 3. Materials and Methods

### 3.1. General

The NMR spectra were measured on a Bruker Avance III (500 MHz for ^1^H-NMR and 125 MHz for ^13^C-NMR, Bruker, Bremen, Germany) spectrometer with TMS as internal standard. Chemical shifts are expressed in ppm with reference to the residual protonated solvent signals (acetone-d6 with δ_H_ 2.05, δ_C_ 206.26, and 29.84) or the internal TMS (0.00). The HR–ESI–MS were recorded on a HR–ESI–MS Bruker microOTOF Q-II (Bremen, Germany). TLC was carried out on precoated silica gel 60 F254 or silica gel 60 RP-18 F254S (Merck, Darmstadt, Germany), and spots were visualized by spraying with 10% H_2_SO_4_ solution followed by heating. Gravity column chromatography was performed with silica gel 60 (0.040–0.063 mm, Himedia, Mumbai, India).

### 3.2. Plant Material

The roots of *Lindera myrrha* were collected from Cu Chi District, Ho Chi Minh City, in July 2016. The botanical sample was authenticated by Dr. Pham Van Ngot, Department of Botany, Faculty of Biology, Ho Chi Minh University of Pedagogy. A voucher specimen (No UP-B05) was deposited in the herbarium of the Department of Organic Chemistry, Faculty of Chemistry, Ho Chi Minh University of Education.

### 3.3. Extraction and Isolation

Roots of *Lindera myrrha* (7.5 kg) were extracted by maceration with MeOH (3 × 20 L) at ambient temperature for 4 h each. The filtrated solution was evaporated to dryness under reduced pressure to obtain a crude extract (420 g). This extract was subsequently reextracted using solvents of increasing polarities, *n*-hexane-EtOAc (1:1) (HA, 72.1 g), and EtOAc (EA, 125.8 g). The latter was applied to normal phase silica gel CC, and isocratically eluted with a solvent system of *n*-hexane-EtOAc-acetone (1:1:1) to afford fraction EA1 (8.1 g). Continuous elution of the column with EtOAc-acetone (1:1), EtOAc-MeOH (8:2), and EtOAc-MeOH (5:5) afforded four fractions, namely EA2 (4.2 g), EA3 (13.6 g), EA4 (7.8 g), and EA5 (40.4 g), respectively.

Fraction EA1 (8.1 g) was rechromatographed on column chromatography, to be isocratically eluted with a CHCl_3_-EtOAc-acetone-AcOH (100:40:25:1) solvent system to afford subfractions EA1.1 (2.03 g), EA1.2 (2.53 g), EA1.3 (1.22 g), and EA1.4 (1.8 g). Among these, subfraction EA1.3 was submitted to Sephadex LH-20 column chromatography, eluted with MeOH to afford three sub-fractions EA1.3.1 (0.7 g), EA1.3.2 (0.3 g), and EA1.3.3 (0.2 g). Fraction EA1.3.2 was further purified by preparative TLC using *n*-hexane-CHCl_3_-EtOAc-acetone-AcOH (1:1:2:2:0.02) as eluent to afford compounds **1** (3.1 mg) and **2** (1.1 mg).

*Myrrhalindenane A* (1). White amorphous solid. ^1^H- and ^13^C-NMR (see Table 1); HRESIMS *m/z* 285.1090 [M + Na]^+^ (calculated for C_15_H_18_O_4_Na, 285.1103).

*Myrrhalindenane B* (2). White amorphous solid. ^1^H- and ^13^C-NMR (see Table 1); HRESIMS *m/z* 301.1047 [M + Na]^+^ (calculated for C_15_H_18_O_5_Na, 301.1052).

### 3.4. Computational Details

All DFT calculations were carried out using Gaussian 09 software package [35]. The stable conformations were optimized at B3LYP/6-311++G(2d,2p) level of theory, as confirmed by the absence of imaginary frequencies at the same level. Theoretical ^13^C-NMR chemical shifts were deduced from the isotropic magnetic shielding tensors by using gauge-independent atomic orbital (GIAO) methodology at B3LYP/6-311+G(d,p) [36,37,38]. The DP4 probabilities were performed using online implementation available from http://www-jmg.ch.cam.ac.uk/tools/nmr/DP4/ [7].

## 4. Conclusions

The investigation of the so-far unstudied terpenic content of *Lindera myrrha* afforded two novel monomeric lindenanes. Despite the elevated number of such metabolites formerly reported to occur in Lauraceae and Chloranthaceae, these two compounds display unusual structural features. Among these, the combination of a native cyclohexanic C ring and of a seco-d cycle, unprecedented within monomeric lindenanes reported so far, is particularly worth being stressed out.

## Figures and Tables

**Table 1:** ^13^C- and ^1^H-NMR spectroscopic data (125/500 MHz) for **1**–**2** in acetone-*d*_6_ (δ in ppm).

	1		2	
	δ_C_	δ_H_ (*J*, Hz)	δ_C_	δ_H_ (*J*, Hz)
1	31.1	1.46, 1H, m	29.1	1.49, 1H, m
2	13.3	1.52, 1H, m	12.3	1.36, 1H, m
		0.71, 1H, m		0.70, 1H, m
3	23.4	2.00, 1H, m	28.7	1.87, 1H, m
4	155.9	-	80.1	-
5	76.5	-	63.8	2.27, 1H, d, 12.0
6	145	6.65, 1H, s	78.4	5.03, 1H, dq, 12.0, 2.0
7	136.1	-	154.6	-
8	197.7	-	197.7	-
8	150	-	148.7	-
9	51.2	2.39, 1H, d, 15.5	56.5	2.67, 1H, d, 16.0
		2.31, 1H, d, 15.5		2.62, 1H, d, 16.0
10	50.8	-	41.4	-
11	38.9	3.50, 1H, q, 7.0	132.1	-
12	174	-	173.9	-
13	16.6	1.26, 3H, d, 7.0	9.8	1.92, 3H, s
14	18.3	1.13, 3H, s	21.9	1.11, 3H, s
15	109	5.17, 1H, s	68.2	3.67, 1H, d, 10.5
		4.99, 1H, s		3.80, 1H, d, 10.5

## Supplementary Materials

The following are available online. ^1^H- and ^13^C-NMR spectra, HMBC spectra, and HRMS spectra for **1** and **2**; atomic coordinates of the lowest-energy conformers of the four candidate diastereoisomers of **1** and **2**.

## References

[1] Zhao M.-L., Song Y., Ni J., Yao X., Tan Y.-H., Xu Z.-F. (2018). Comparative chloroplast genomics and phylogenetics of nine *Lindera* species (Lauraceae). Sci. Rep..

[2] Cao Y., Xuan B., Peng B., Li C., Chai X., Tu P. (2016). The genus *Lindera*: a source of structurally diverse molecules having pharmacological significance. Phytochem. Rev..

[3] Tanaka H., Ichino K., Ito K. (1985). A novel flavanone, linderatone, from *Lindera umbellata*. Chem. Pharm. Bull. (Tokyo).

[4] Phan B.-H., Seguin E., Tillequin F., Koch M. (1994). Aporphine alkaloids from *Lindera myrrha*. Phytochemistry.

[5] Duong T.-H., Nguyen V.-K., Nguyen-Pham K.-T., Sichaem J., Nguyen H.-D. (2019). Lindermyrrhin, a novel 3,4-dihydroisocoumarin from *Lindera myrrha* roots. Nat. Prod. Res..

[6] Hayashi N., Komae H. (1980). Chemistry and distribution of sesquiterpene furans in Lauraceae. Biochem. Syst. Ecol..

[7] Smith S.G., Goodman J.M. (2010). Assigning Stereochemistry to Single Diastereoisomers by GIAO NMR Calculation: The DP4 Probability. J. Am. Chem. Soc..

[8] Wu B., He S., Pan Y. (2007). Sesquiterpenoid with new skeleton from *Chloranthus henryi*. Tetrahedron Lett..

[9] Du B., Huang Z., Wang X., Chen T., Shen G., Fu S., Liu B. (2019). A unified strategy toward total syntheses of lindenane sesquiterpenoid [4 + 2] dimers. Nat. Commun..

[10] Saito Y., Ichihara M., Okamoto Y., Gong X., Kuroda C., Tori M. (2014). Twelve new compounds from *Ligularia melanothyrsa*; isolation of melanothyrsins A–E, normelanothyrsin A, and other eremophilane sesquiterpenoids. Tetrahedron.

[11] Shi Y.-S., Liu Y.-B., Ma S.-G., Li Y., Qu J., Li L., Yuan S.-P., Hou Q., Li Y.-H., Jiang J.-D. (2015). Bioactive Sesquiterpenes and Lignans from the Fruits of *Xanthium sibiricum*. J. Nat. Prod..

[12] Ishiyama H., Hashimoto A., Fromont J., Hoshino Y., Mikami Y., Kobayashi J. (2005). Halichonadins A–D, new sesquiterpenoids from a sponge *Halichondria* sp.. Tetrahedron.

[13] Xu Y.-J. (2013). Phytochemical and Biological Studies of *Chloranthus* Medicinal Plants. Chem. Biodivers..

[14] Kawabata J., Mizutani J. (1989). Shizukanolides D, E and F, Novel Lindenanolides from *Chloranthus* spp. (Chloranthaceae). Agric. Biol. Chem..

[15] Hu X., Yang J., Xu X. (2009). Three Novel Sesquiterpene Glycosides of *Sarcandra glabra*. Chem. Pharm. Bull. (Tokyo).

[16] Yue G., Yang L., Yuan C., Du B., Liu B. (2012). Total syntheses of lindenane-type sesquiterpenoids: (±)-chloranthalactones A, B, F, (±)-9-hydroxy heterogorgiolide, and (±)-shizukanolide E. Tetrahedron.

[17] Fenlon T.W., Jones M.W., Adlington R.M., Lee V. (2013). Synthesis of *rac*-Lindenene via a thermally induced cyclopropanation reaction. Org. Biomol. Chem..

[18] Wu B., Chen J., Qu H., Cheng Y. (2008). Complex Sesquiterpenoids with Tyrosinase Inhibitory Activity from the Leaves of *Chloranthus tianmushanensis*. J. Nat. Prod..

[19] Cao C.-M., Peng Y., Shi Q.-W., Xiao P.-G. (2008). Chemical Constituents and Bioactivities of Plants of Chloranthaceae. Chem. Biodivers..

[20] Wang A.-R., Song H.-C., An H.-M., Huang Q., Luo X., Dong J.-Y. (2015). Secondary Metabolites of Plants from the Genus *Chloranthus*: Chemistry and Biological Activities. Chem. Biodivers..

[21] He X.-F., Yin S., Ji Y.-C., Su Z.-S., Geng M.-Y., Yue J.-M. (2010). Sesquiterpenes and Dimeric Sesquiterpenoids from *Sarcandra glabra*. J. Nat. Prod..

[22] Ni G., Zhang H., Liu H.-C., Yang S.-P., Geng M.-Y., Yue J.-M. (2013). Cytotoxic sesquiterpenoids from *Sarcandra glabra*. Tetrahedron.

[23] Kouno I., Hirai A., Fukushige A., Jiang Z.-H., Tanaka T. (2001). New Eudesmane Sesquiterpenes from the Root of *Lindera strychnifolia*. J. Nat. Prod..

[24] Zhang C., Nakamura N., Tewtrakul S., Hattori M., Sun Q., Wang Z., Fujiwara T. (2002). Sesquiterpenes and Alkaloids from Lindera chunii and Their Inhibitory Activities against HIV-1 Integrase. Chem. Pharm. Bull..

[25] Gan L.-S., Zheng Y.-L., Mo J.-X., Liu X., Li X.-H., Zhou C.-X. (2009). Sesquiterpene Lactones from the Root Tubers of *Lindera aggregata*. J. Nat. Prod..

[26] Wang L.-J., Xiong J., Liu S.-T., Liu X.-H., Hu J.-F. (2014). Sesquiterpenoids from *Chloranthus henryi* and Their Anti-neuroinflammatory Activities. Chem. Biodivers..

[27] Kawabata J., Fukushi Y., Tahara S., Mizutani J. (1990). Shizukaol a, a sesquiterpene dimer from *Chloranthus japonicus*. Phytochemistry.

[28] Kawabata J., Mizutani J. (1992). Dimeric sesquiterpenoid esters from *Chloranthus serratus*. Phytochemistry.

[29] Kawabata J., Fukushi E., Mizutani J. (1995). Sesquiterpene dimers from *Chloranthus japonicus*. Phytochemistry.

[30] Yang S.-P., Gao Z.-B., Wang F.-D., Liao S.-G., Chen H.-D., Zhang C.-R., Hu G.-Y., Yue J.-M. (2007). Chlorahololides A and B, Two Potent and Selective Blockers of the Potassium Channel Isolated from *Chloranthus holostegius*. Org. Lett..

[31] Yang S.-P., Gao Z.-B., Wu Y., Hu G.-Y., Yue J.-M. (2008). Chlorahololides C–F: a new class of potent and selective potassium channel blockers from *Chloranthus holostegius*. Tetrahedron.

[32] Kim S.-Y., Kashiwada Y., Kawazoe K., Murakami K., Sun H.-D., Li S.-L., Takaishi Y. (2009). Spicachlorantins C–F, hydroperoxy dimeric sesquiterpenes from the roots of *Chloranthus spicatus*. Tetrahedron Lett..

[33] Yan H., Ba M.-Y., Li X.-H., Guo J.-M., Qin X.-J., He L., Zhang Z.-Q., Guo Y., Liu H.-Y. (2016). Lindenane sesquiterpenoid dimers from *Chloranthus japonicus* inhibit HIV-1 and HCV replication. Fitoterapia.

[34] Yuan C., Du B., Deng H., Man Y., Liu B. (2017). Total Syntheses of Sarcandrolide J and Shizukaol D: Lindenane Sesquiterpenoid [4+2] Dimers. Angew. Chem. Int. Ed..

[35] Frisch M.J., Trucks G.W., Schlegel H.B., Scuseria G.E., Robb M.A., Cheeseman J.R., Scalmani G., Barone V., Mennucci B., Petersson G.A. (2013). Gaussian09 Revision D.01.

[36] Konstantinov I.A., Broadbelt L.J. (2011). Regression formulas for density functional theory calculated 1H and 13C NMR chemical shifts in toluene-d_8_. J. Phys. Chem. A.

[37] Ditchfield R. (1974). Self-consistent perturbation theory of diamagnetism: I. A gauge-invariant LCAO method for NMR chemical shifts. Mol. Phys..

[38] Wolinski K., Hinton J.F., Pulay P. (1990). Efficient implementation of the gauge-independent atomic orbital method for NMR chemical shift calculations. J. Am. Chem. Soc..

