# Supplementary material for: Atypical Lindenane-Type Sesquiterpenes from Lindera myrrha
Source: Molecules. 2020 Apr 16;25(8):1830. doi: 10.3390/molecules25081830 (PMC7221893; doi:10.3390/molecules25081830)
Supplement: Supplementary file 1 [file molecules-25-01830-s001.pdf]

Supplementary Material of

Atypical Lindenane-type Sesquiterpenes from *Lindera myrrha*

Van-Giau Vo[1,2], Thuc-Huy Duong[3], Mehdi A. Beniddir[4], Nguyen T. Trung[5], Cam-Tu D. Phan[5], Van-Kieu Nguyen[6,7], Quynh-Loan Le[8], Hoang-Dung Nguyen[8,9*], and Pierre Le Pogam[4*]

[1] Bionanotechnology Research Group, Ton Duc Thang University, Ho Chi Minh City 700000, Vietnam
[2] Faculty of Pharmacy, Ton Duc Thang University, Ho Chi Minh City 700000, Vietnam
[3] Department of Chemistry, University of Education, 280 An Duong Vuong Street, District 5, Ho Chi Minh City, Vietnam.
[4] Équipe "Chimie des Substances Naturelles", BioCIS, Université Paris-Saclay, CNRS, 5 Rue J.-B. Clément, 92290 Châtenay-Malabry, France.
[5] Laboratory of Computational Chemistry and Modelling (LCCM), Quy Nhon University, 55100, Viet Nam
[6] Institute of Fundamental and Applied Sciences, Duy Tan University, Ho Chi Minh City 700000, Vietnam.
[7] Faculty of Natural Sciences, Duy Tan University, Da Nang, 550000, Vietnam.
[8] Institute of Tropical Biology, Vietnam Academy of Science and Technology, Ho Chi Minh City, Vietnam
[9] NTT-High tech Institute, Nguyen Tat Thanh University, Ho Chi Minh City, Vietnam Graduate University of Science and Technology, Vietnam Academy of Science and Technology, 18 Hoang Quoc Viet, Cau Giay, Ha Noi,Vietnam

*Correspondence: dung0018034@yahoo.com (H.-D. N.), pierre.le-pogam-alluard@universite-paris-saclay.fr (P.L.P.).

Summary of the Supporting Material content

S1 HRESIMS of **1**

S2 ^{1}H-NMR spectrum of **1** (500 MHz, Acetone-d_6)

S3 ^{13}C-NMR spectrum of **1** (125 MHz, Acetone-d_6)

S4 COSY spectrum of **1** (500 MHz, Acetone-d_6)

S5 HSQC spectrum of **1** (500/125 MHz, Acetone-d_6)

S6 HMBC spectrum of **1** (500/125 MHz, Acetone-d_6)

S7 NOESY spectrum of **1** (500 MHz, Acetone-d_6)

S8 HRESIMS of **2**

S9 ^{1}H-NMR spectrum of **2** (500 MHz, Acetone-d_6)

S10 ^{13}C-NMR spectrum of **2** (125 MHz, Acetone-d_6)

S11 COSY spectrum of **2** (500/125 MHz, Acetone-d_6)

S12 HSQC spectrum of **2** (500/125 MHz, Acetone-d_6)

S13 HMBC spectrum of **2** (500/125 MHz, Acetone-d_6)

S14 Atomic coordinates (Ångstroms) of the lowest-energy conformers of candidate structures of myrrhalindenane A (**1A**-**1D**).

S15 Atomic coordinates (Ångstroms) of the lowest-energy conformers of candidate structures of myrrhalindenane B (**2A**-**2D**).

S1. HRESIMS of **1**

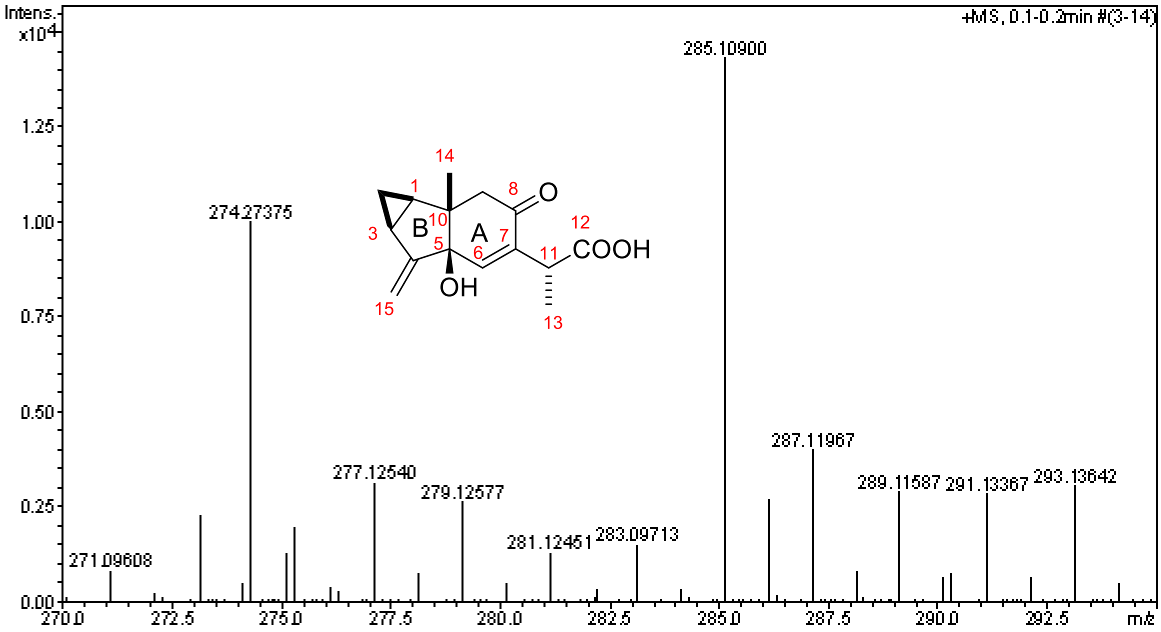

S2. ^{1}H-NMR spectrum of **1** (500 MHz, Acetone-d6)

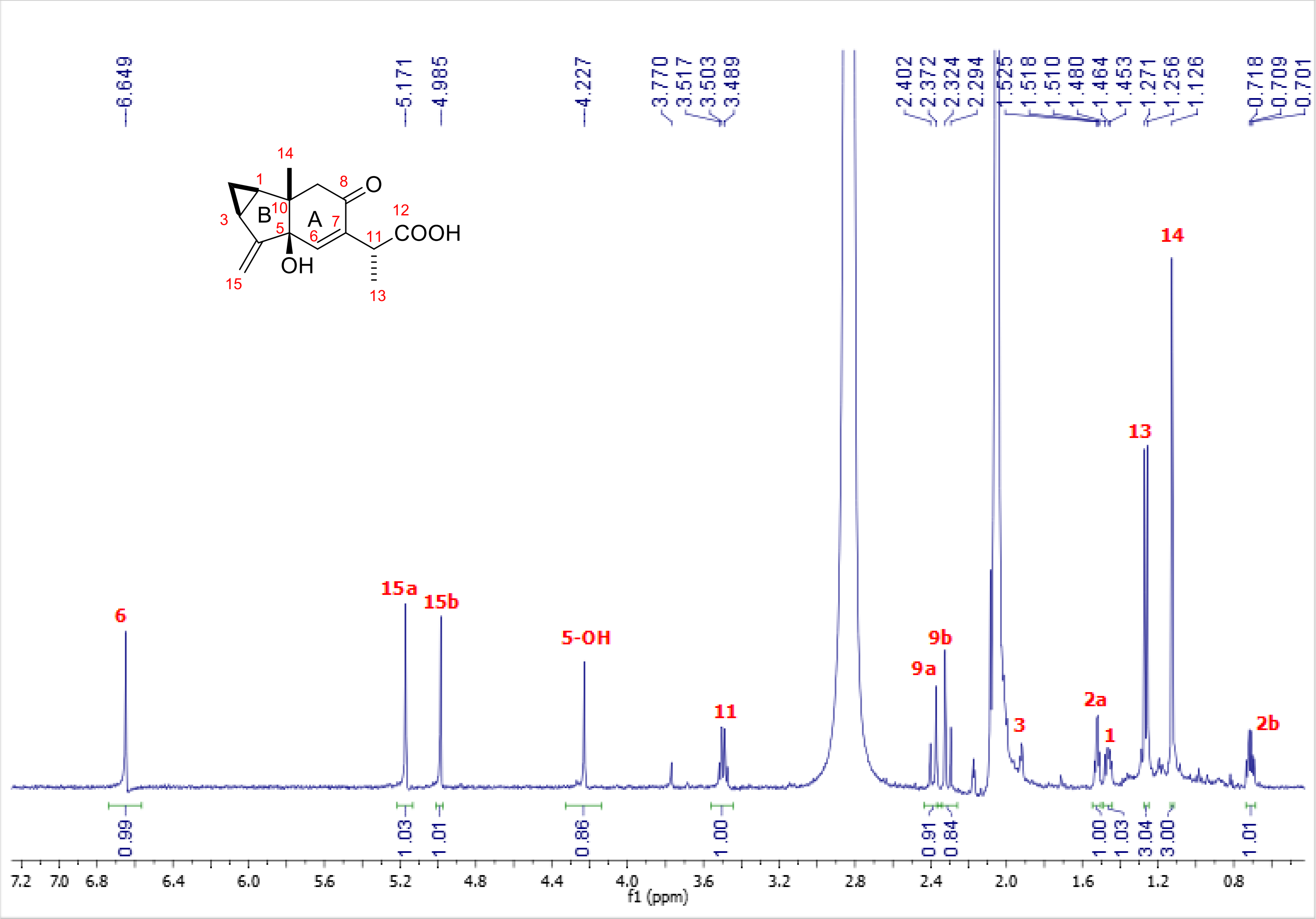

S3. ^{13}C-NMR spectrum of **1** (125 MHz, Acetone-d6)

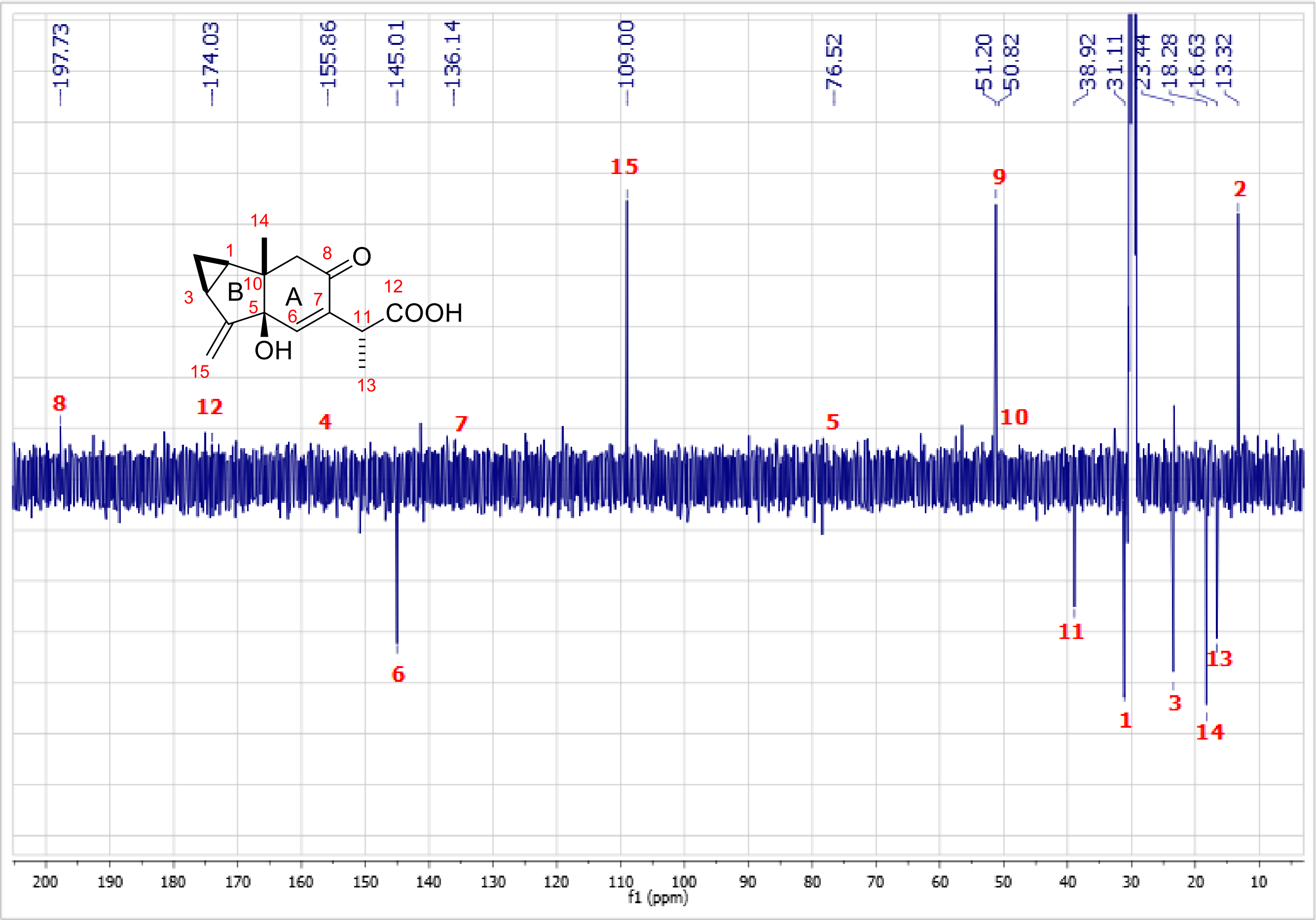

S4. COSY spectrum of **1** (500/125 MHz, Acetone-d6)

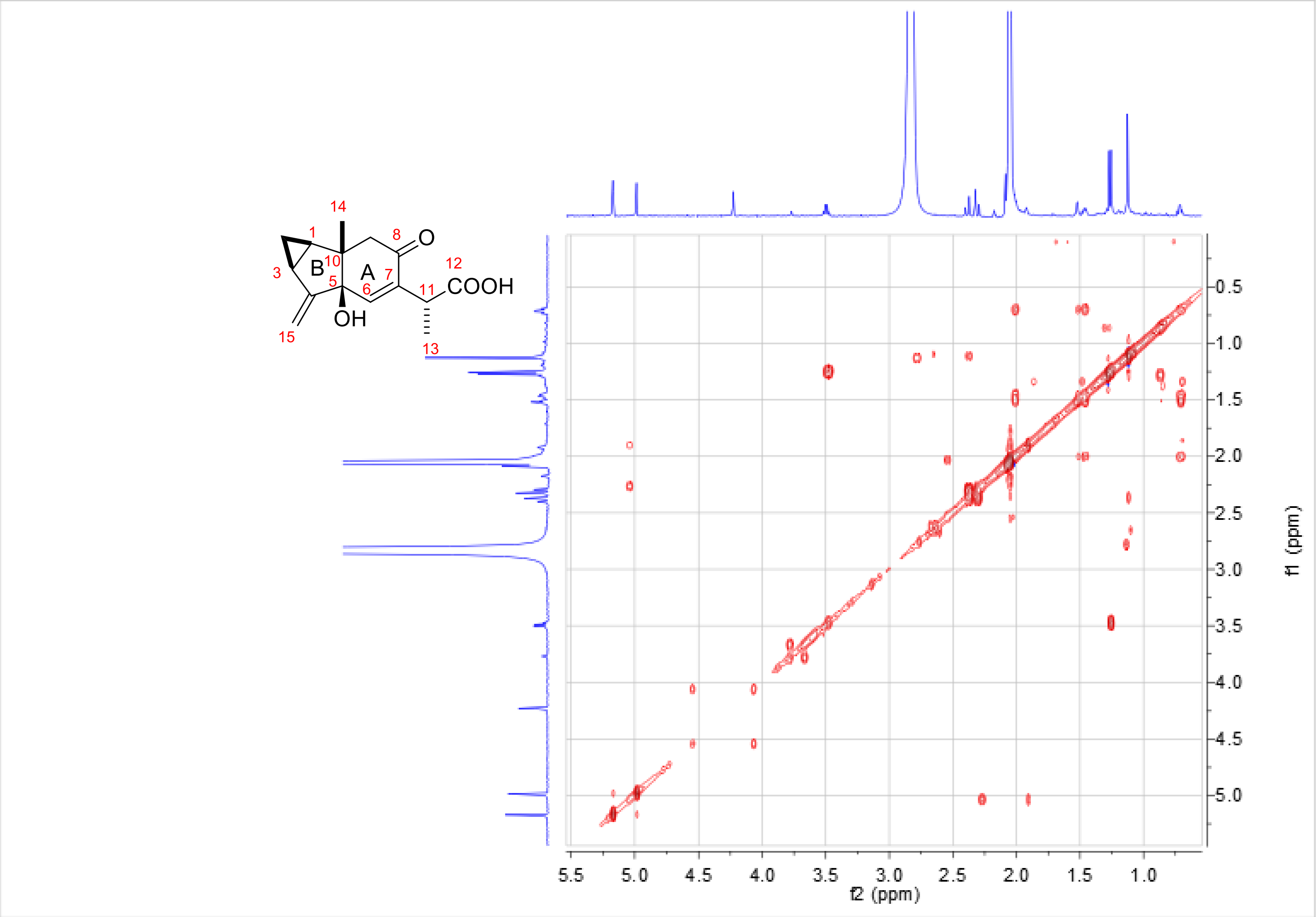

S5. HSQC spectrum of **1** (500/125 MHz, Acetone-d6)

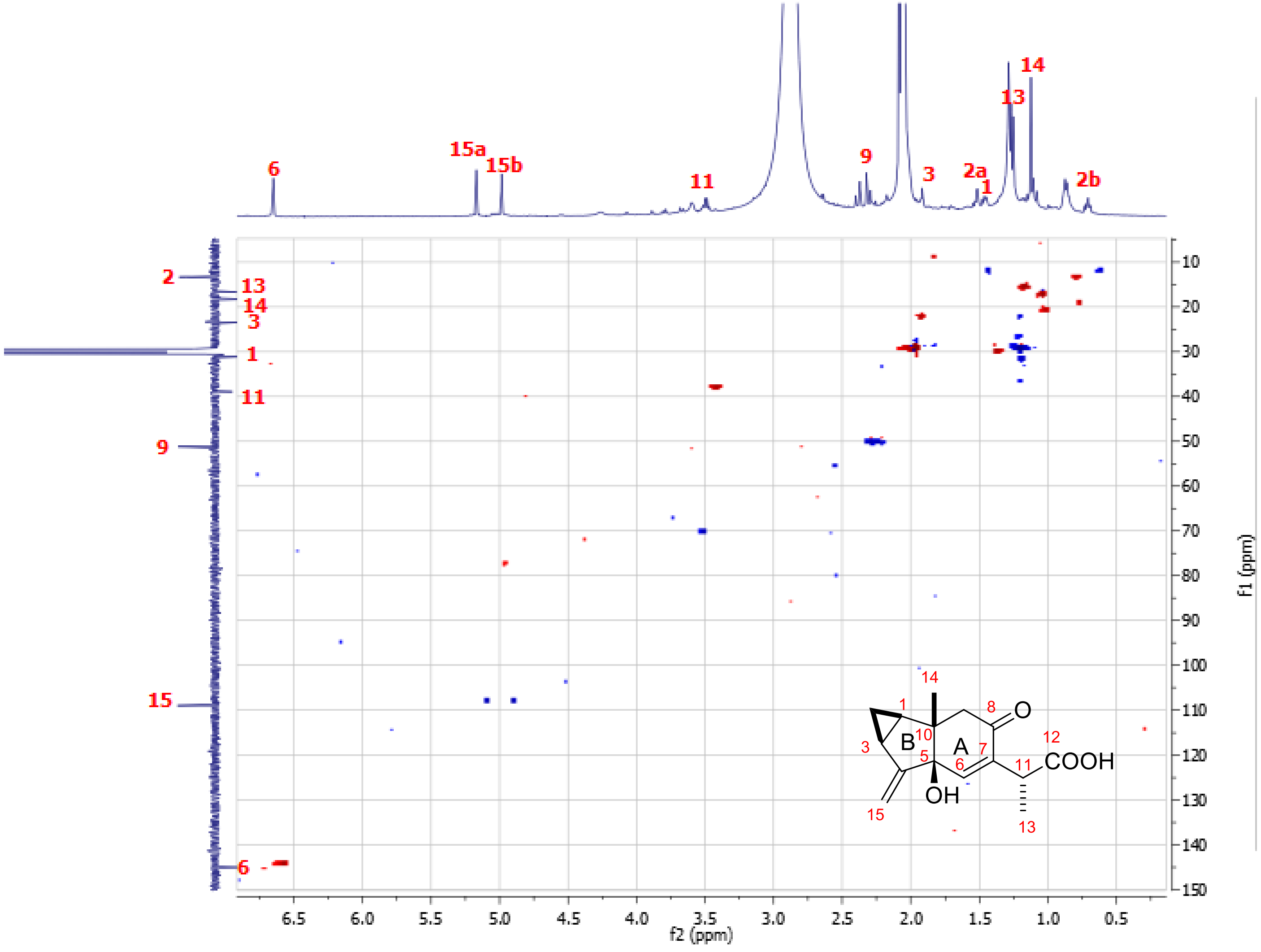

S6. HMBC spectrum of **1** (500/125 MHz, Acetone-d6)

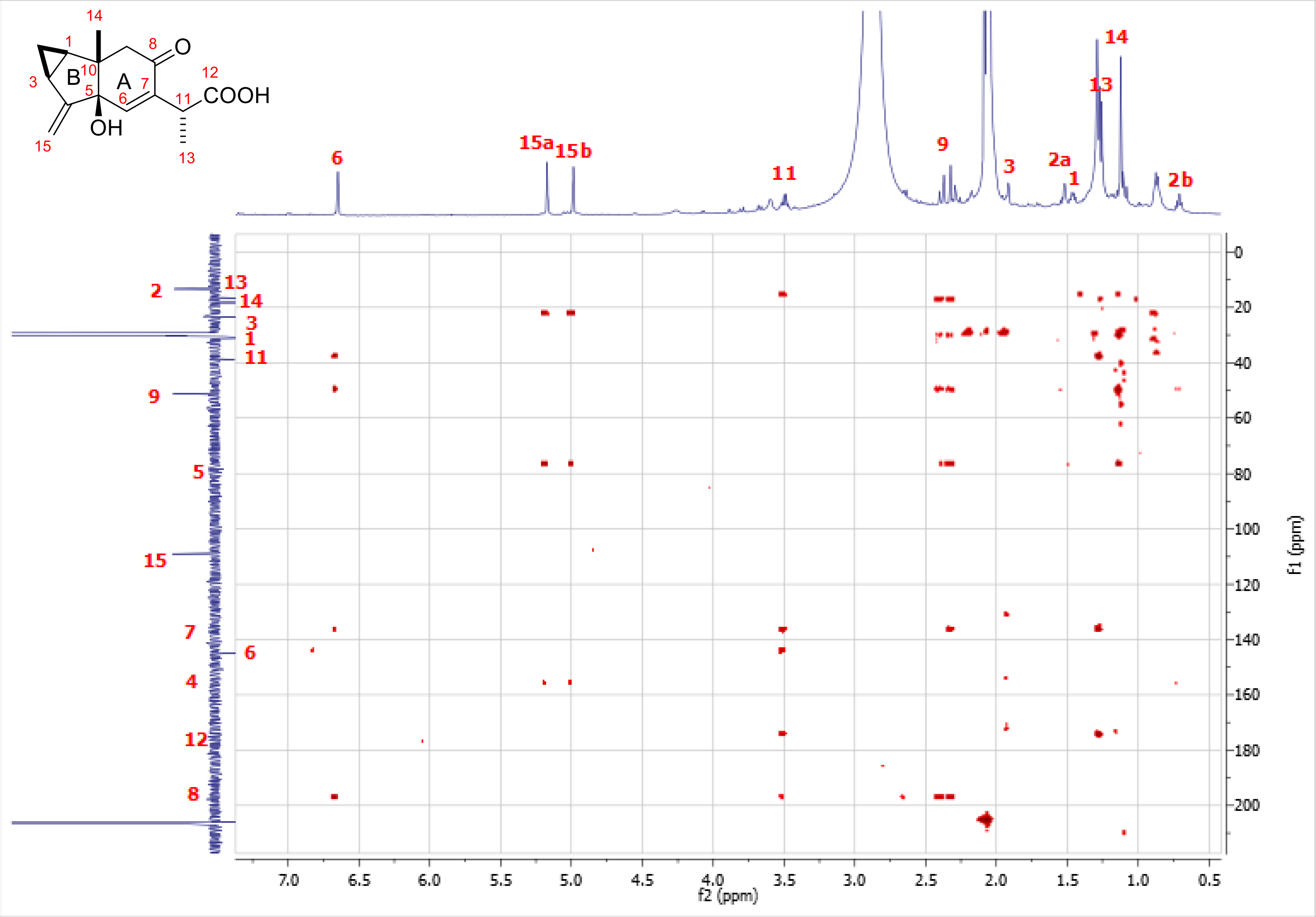

S7. NOESY spectrum of **1** (125 MHz, Acetone-d6)

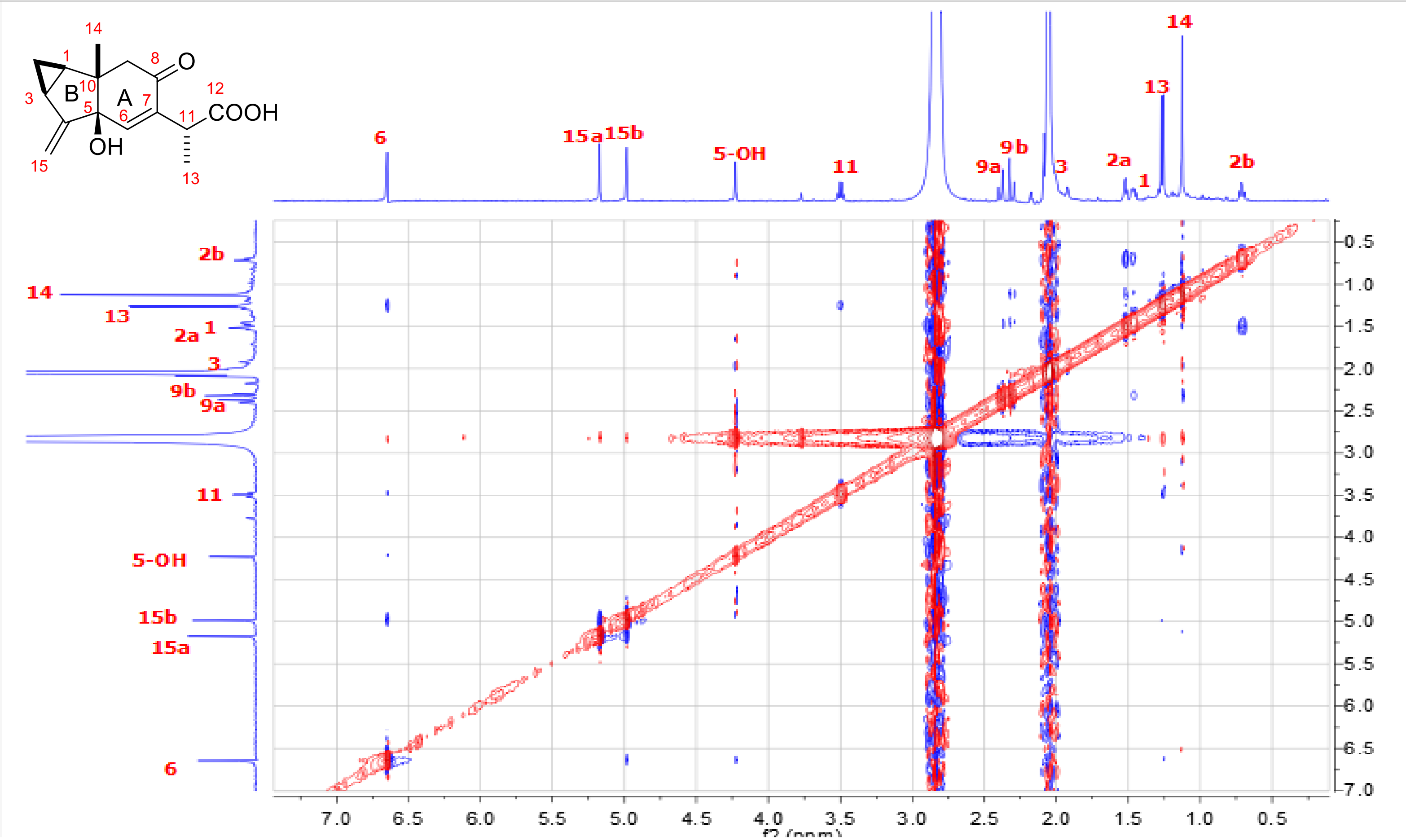

S8. HRESIMS of **2**

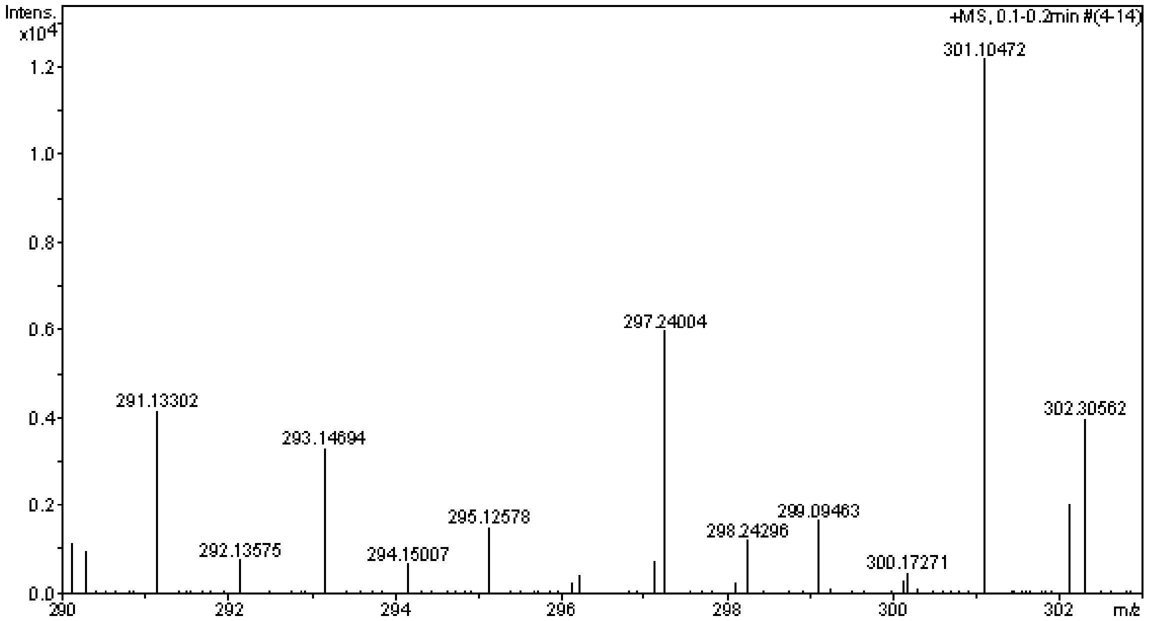

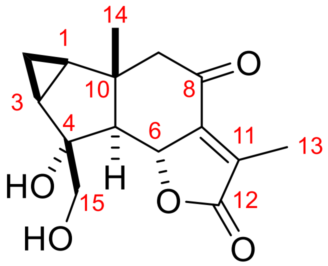

S9. ¹H-NMR spectrum of **2** (500 MHz, Acetone-d6)

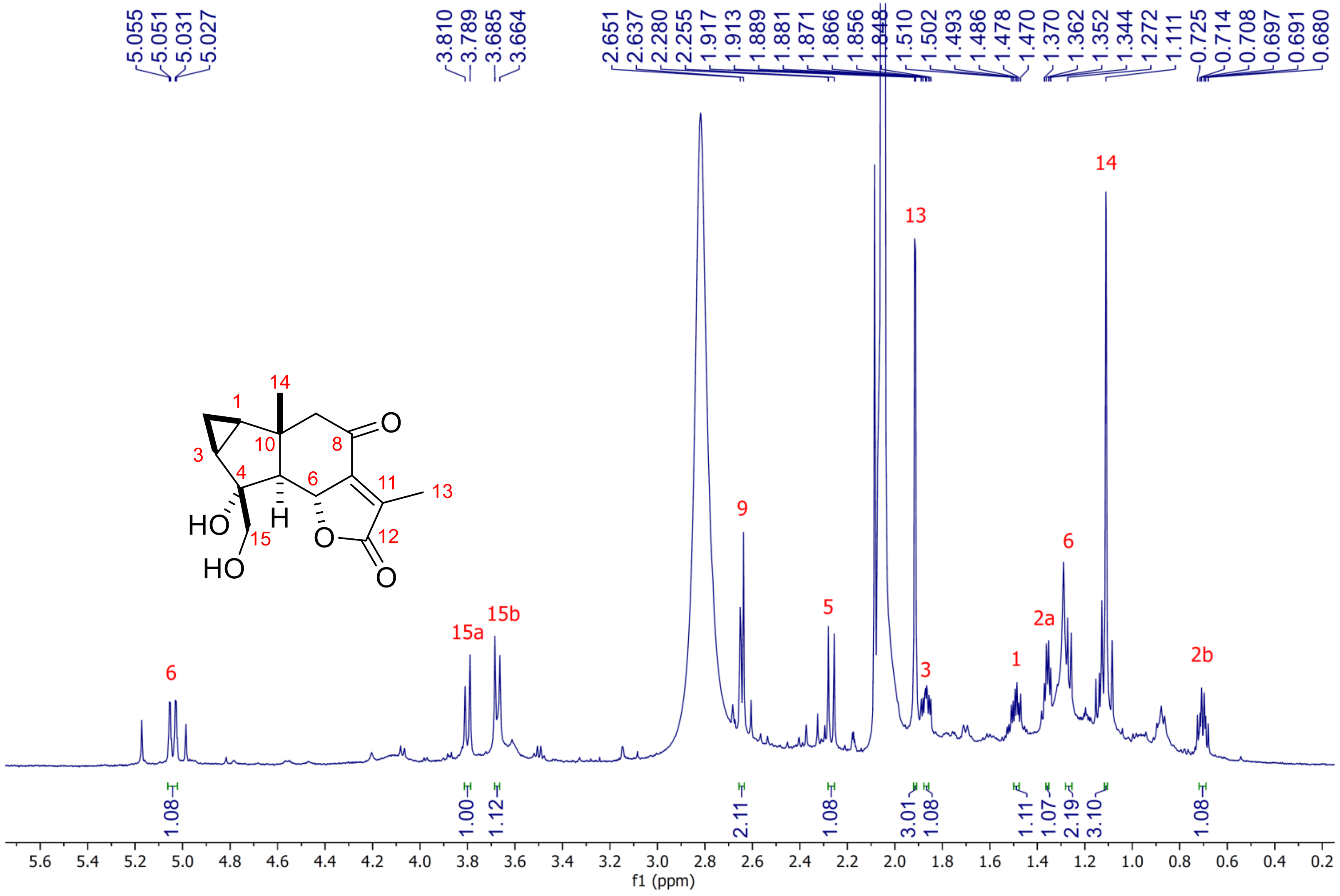

S10. ^{13}C-NMR spectrum of **2** (125 MHz, Acetone-d6)

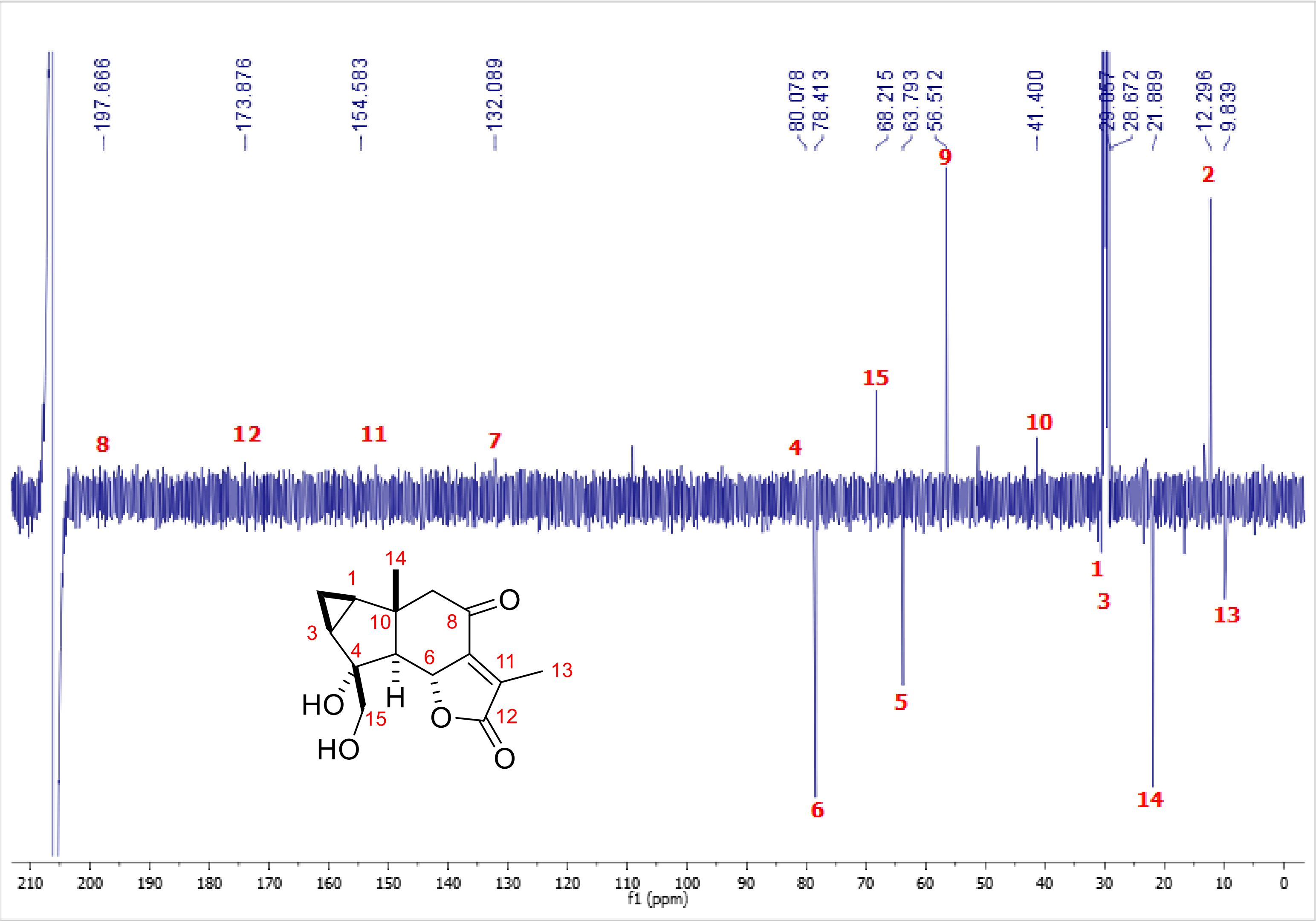

S11. COSY spectrum of **2** (500/125 MHz, Acetone-d6)

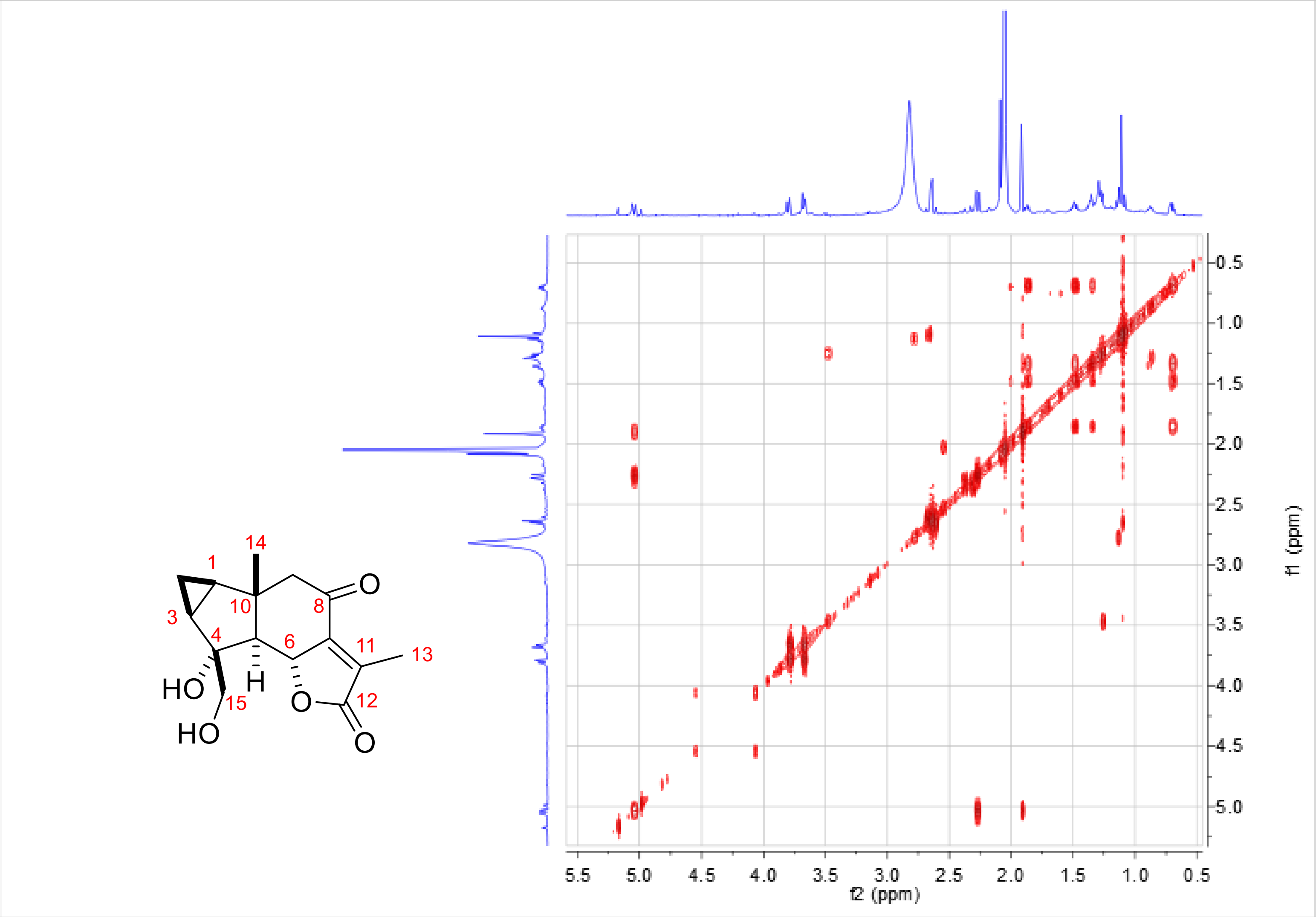

S12. HSQC spectrum of **2** (500/125 MHz, Acetone-d6)

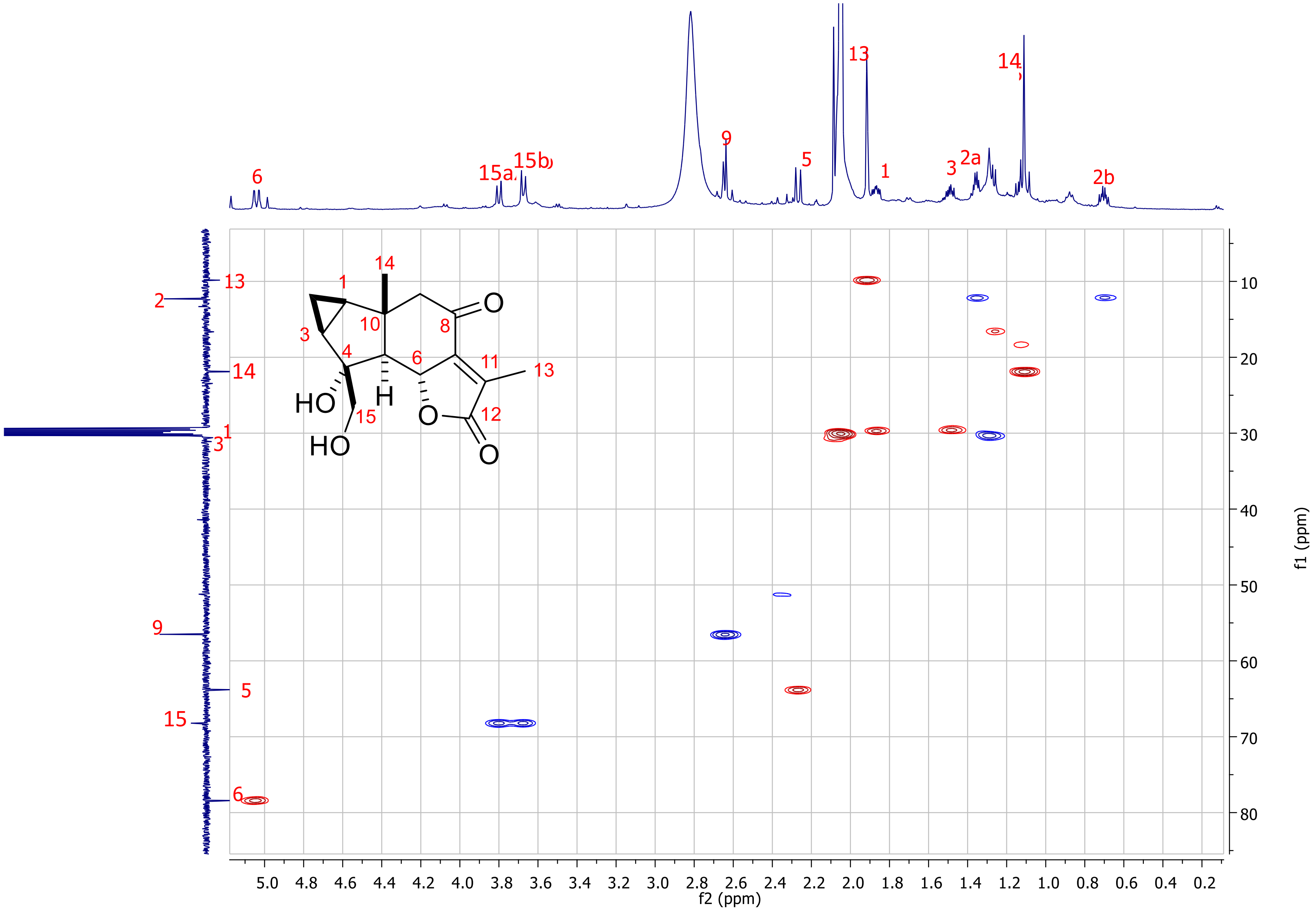

S13. HMBC spectrum of **2** (500/125 MHz, Acetone-d6)

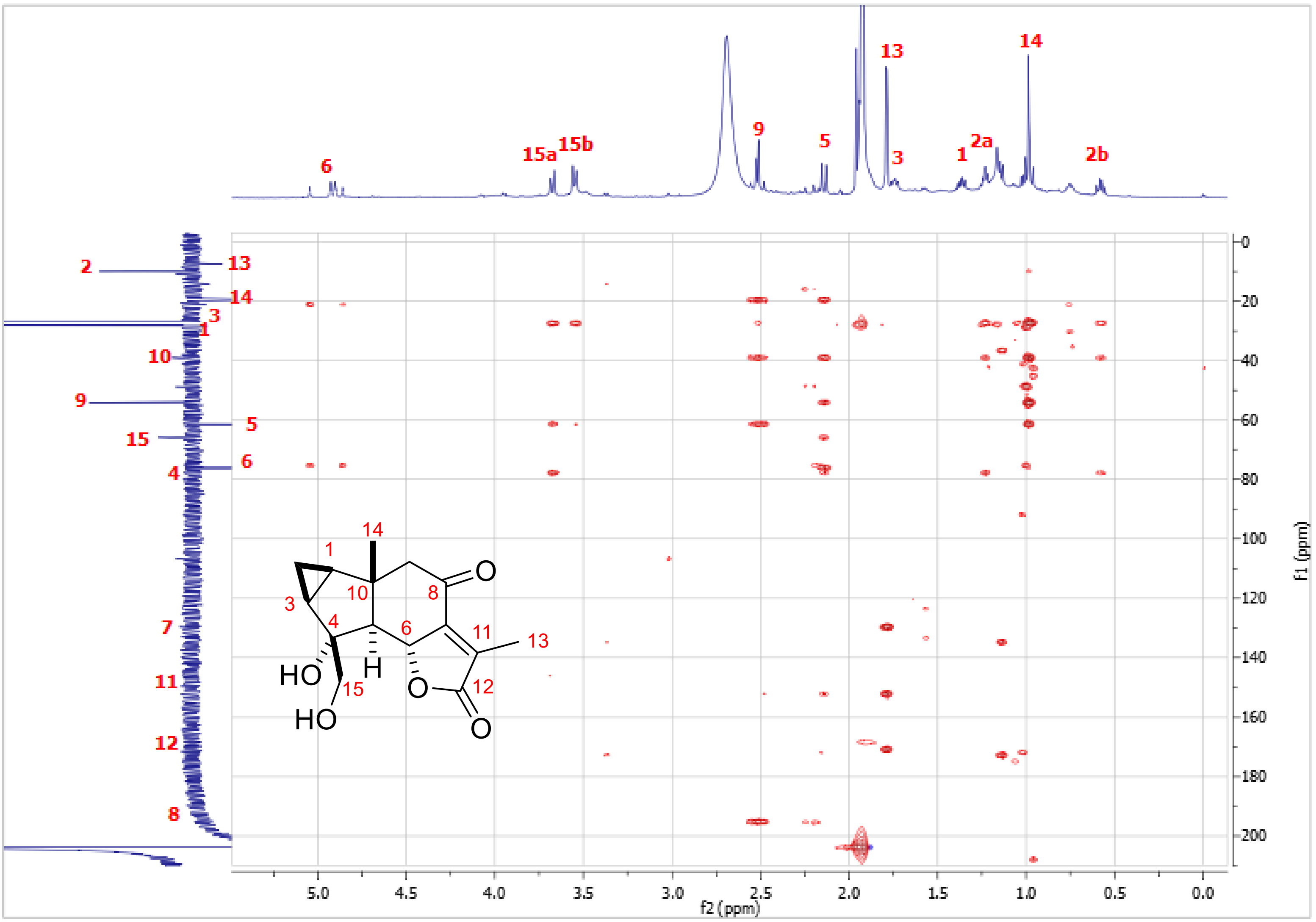

S14. Atomic coordinates (Ångstroms) of the lowest-energy conformers of candidate structures of myrrhalindenane A (**1A**-**1D**).

1A

C	1.18951200	0.48189900	-0.07867600
C	0.41226700	-0.45490100	0.47647700
C	-1.74854100	-1.53047800	-0.38434400
H	0.87891800	-1.26471100	1.02749600
C	2.70663200	0.52007100	0.05869700
H	3.08186500	1.11545600	-0.77070500
C	0.56108000	1.59187600	-0.84181300
O	1.20857600	2.53617900	-1.25400300
C	-1.75199000	0.87704600	0.04000100
C	-1.09406700	-0.46457800	0.50578400
C	-1.86529600	1.92215900	1.15450100
H	-0.88733700	2.19348800	1.55350800
H	-2.32729800	2.82827700	0.76002700
H	-2.47152400	1.55600700	1.97812100
O	-1.47626100	-0.69725900	1.88351500
H	-1.34334100	-1.63286100	2.06913100
C	-3.10985100	0.45956100	-0.53279400
H	-3.56933500	1.13751000	-1.23970300
C	-3.07594000	-1.03238100	-0.81499300
H	-3.52434800	-1.46092500	-1.69928600
C	-3.99850500	-0.45296400	0.25036400
H	-5.04862300	-0.44065900	-0.00511600
H	-3.77973200	-0.64858300	1.28760700
C	-0.91573200	1.44245500	-1.13145100
H	-0.99204300	0.76752800	-1.99030200
H	-1.30170700	2.41082200	-1.44625100
C	3.27604500	-0.87790300	-0.07124900
O	3.67213000	-1.15182100	-1.33706700
H	3.99618200	-2.06444600	-1.33944600
O	3.36591200	-1.68897500	0.81791300
C	3.13819600	1.17141500	1.38252200
H	4.22342000	1.25964800	1.43344700
H	2.71161800	2.17017800	1.45534100
H	2.80156100	0.57910700	2.23097900
C	-1.17936700	-2.67914500	-0.74342000
H	-1.70574000	-3.39759900	-1.35726700
H	-0.17204800	-2.93802200	-0.44808000

1B

C	1.21640700	0.42543600	-0.04321500
C	0.41380200	-0.64362100	0.04085500
C	-2.08593300	-1.52238600	0.04541900
H	0.80592100	-1.61148900	0.32583400
C	2.70379200	0.40748600	0.27979800
C	0.66530200	1.76637100	-0.45346400
O	1.41047800	2.70984400	-0.63125000
C	-1.63317400	0.83968900	0.08405700
C	-1.49230100	1.06706300	1.60627300
H	-0.47880800	1.35085000	1.87850600
H	-2.15762400	1.87318300	1.91586400
H	-1.74086900	0.18161200	2.18889500
C	-3.11362300	0.65269700	-0.32903400
H	-3.51247000	1.28065300	-1.11334700
C	-3.38869800	-0.85224800	-0.31824300
H	-4.01184800	-1.32201300	-1.06802200
C	-4.07460900	0.06355100	0.66712900
H	-5.12689900	0.23866700	0.49004500
H	-3.80781300	0.00971500	1.71049900
C	-0.84657200	1.93693600	-0.63693300
H	-1.03119200	1.91473400	-1.71125100
H	-1.10263300	2.93462500	-0.28028800
C	-1.02011000	-0.52420200	-0.37337800
O	-0.94640000	-0.57257100	-1.82737700
H	-1.83136500	-0.73362000	-2.17166900
C	3.30743600	-0.93556000	-0.07701000
O	3.84479700	-0.93889300	-1.31862000
H	4.17336900	-1.83507000	-1.48187800
O	3.31554800	-1.91404800	0.62963500
C	2.96779700	0.75319600	1.75471600
H	2.54469600	-0.00624400	2.40931900
H	4.03814400	0.81570400	1.95002700
H	2.52452700	1.71849100	1.99303100
H	3.16764500	1.16287900	-0.35119600
C	-1.91348300	-2.72756400	0.57117300
H	-0.92881600	-3.12677700	0.77293400
H	-2.75554700	-3.36163100	0.81473700

1C

C	-1.18918000	0.40777200	0.24715800
C	-0.41296400	-0.63173500	-0.07964600
C	1.90758800	-1.29964800	0.77255900
H	-0.87388800	-1.59588600	-0.26575700
C	-2.70235600	0.34241600	0.39830100
C	-0.55927700	1.72400300	0.53648600
O	-1.22224000	2.72652300	0.72669400
C	1.68075900	0.84333300	-0.38194300
C	1.07554600	-0.59857600	-0.31054200
C	1.58521300	1.47936600	-1.77244200
H	0.55049900	1.57152000	-2.10421700
H	2.01759200	2.48061300	-1.74598100
H	2.12161700	0.89172000	-2.51203200
O	1.31031500	-1.24857600	-1.58380500
H	1.23822100	-2.19853500	-1.44319300
C	3.12361700	0.67849800	0.10580500
H	3.60942500	1.56300300	0.49583400
C	3.23464300	-0.64418400	0.84360400
H	3.81318300	-0.75142200	1.74942100
C	3.98165000	-0.40568400	-0.46310400
H	5.05038300	-0.27606900	-0.36740200
H	3.65916300	-0.92942800	-1.34826800
C	0.94769500	1.72443100	0.65532500
H	1.17452200	1.36085900	1.66303600
H	1.29452400	2.75504900	0.59768000
C	-3.10997300	0.04325400	1.85019600
H	-2.66879900	0.78273800	2.51602500
H	-4.19295400	0.08752200	1.96380300
H	-2.77490200	-0.94848200	2.14739700
C	1.47307400	-2.29104700	1.54758500
H	2.12079800	-2.75462600	2.27951200
H	0.46082600	-2.66626900	1.48833500
C	-3.29057700	-0.67829400	-0.55329000
O	-3.38633000	-1.86305300	-0.34334600
O	-3.69730800	-0.11777200	-1.71604000
H	-4.02684800	-0.83601800	-2.27586400
H	-3.08160700	1.32331400	0.11733000

1D

C	-1.23612800	0.43470100	0.20790500
C	-0.40352800	-0.59008300	0.43167300
C	2.11631500	-1.41063400	0.44067500
H	-0.78572300	-1.59117400	0.58915800
C	-2.75267600	0.29721900	0.17834700
C	-0.70344900	1.83185100	0.03343100
O	-1.46571200	2.77567900	-0.03873300
C	1.55173300	0.76600600	-0.41820100
C	1.21759500	0.41110500	-1.88489500
H	0.16647100	0.57358200	-2.10965000
H	1.80280600	1.04319600	-2.55295000
H	1.43650500	-0.62775900	-2.12621200
C	3.07842700	0.74361900	-0.16049600
H	3.54025600	1.61984700	0.27246300
C	3.41997700	-0.65535900	0.35713900
H	4.14998600	-0.80986000	1.14093800
C	3.93798500	-0.18009500	-0.97923700
H	4.99462000	0.04640800	-1.01894900
H	3.54943600	-0.62366700	-1.88206600
C	0.80970500	2.05537200	-0.05713400
H	1.12576300	2.43511600	0.91458600
H	0.97329300	2.84771600	-0.78778100
C	1.06218100	-0.32700200	0.58792600
O	1.16207900	0.17369300	1.95252700
H	2.08754300	0.15101000	2.21833500
C	-3.35042600	0.34245600	1.59256400
H	-3.01308900	-0.50856500	2.18036600
H	-3.04355800	1.26015100	2.09047800
H	-4.43929200	0.32161200	1.55097700
C	1.93943600	-2.72490100	0.43611400
H	0.95795800	-3.16922800	0.53069100
H	2.77528500	-3.40561100	0.34414900
C	-3.13840400	-0.97750600	-0.54386500
O	-3.30516600	-2.05826800	-0.03525900
O	-3.26103300	-0.78541100	-1.88090000
H	-3.47690200	-1.64581000	-2.26900300
H	-3.13515800	1.13699700	-0.39830100

S15 Atomic coordinates (Ångstroms) of the lowest-energy conformers of candidate structures of myrrhalindenane B (**2A**-**2D**).

2A

C	1.743484	1.858031	0.016413
O	2.678695	2.618113	0.154998
C	-0.790076	1.504597	0.033040
C	-0.925346	1.862364	1.531369
H	0.036918	1.861755	2.044674
H	-1.336012	2.867731	1.623589
H	-1.581507	1.188604	2.077511
C	-2.110810	1.712263	-0.737354
H	-2.124512	2.461455	-1.517090
C	-2.765581	0.359405	-0.944663
H	-3.254578	0.114160	-1.875189
C	-3.410125	1.376975	-0.050042
H	-4.302564	1.853492	-0.430843
H	-3.428837	1.223952	1.017962
C	0.387171	2.330904	-0.518622
H	0.434674	2.235665	-1.608332
H	0.293942	3.392678	-0.291514
C	-0.489854	0.001446	-0.266758
C	2.435773	-1.816818	0.098136
O	1.104852	-1.808257	0.420822
O	3.060866	-2.825623	-0.070919
H	-0.127948	-0.030588	-1.297820
C	-1.837329	-0.748866	-0.392111
O	-1.648566	-1.750568	-1.391788
H	-2.402878	-2.349633	-1.333936
C	-2.322856	-1.442488	0.879196
H	-1.551894	-2.141552	1.208894
C	0.662939	-0.456561	0.612585
H	0.407135	-0.372376	1.670565
C	1.864106	0.392267	0.267924
C	2.904891	-0.402683	-0.018664
H	-2.520383	-0.726213	1.679091
O	-3.523405	-2.146126	0.539225
H	-3.687630	-2.826999	1.195636
C	4.288726	-0.091394	-0.460793
H	4.553218	-0.697076	-1.327821
H	4.408142	0.965131	-0.681584
H	4.994500	-0.357133	0.329572

2B

C	-1.433613	-2.051944	-0.043449
O	-2.280208	-2.908724	0.091897
C	1.046783	-1.448942	-0.020424
C	1.234649	-1.871662	1.453769
H	0.280008	-1.969312	1.973411
H	1.715967	-2.849330	1.487958
H	1.842082	-1.166156	2.011956
C	2.370829	-1.467967	-0.814567
H	2.429987	-2.122796	-1.673464
C	2.894144	-0.044132	-0.896335
H	3.325405	0.340248	-1.810782
C	3.655406	-1.085245	-0.128120
H	4.571850	-1.443216	-0.575838
H	3.689226	-1.001000	0.945930
C	-0.048096	-2.349909	-0.620922
H	-0.122584	-2.187244	-1.701804
H	0.155433	-3.409242	-0.467014
C	0.618054	0.041952	-0.215725
C	-2.499761	1.526453	0.083552
O	-1.173799	1.647833	0.439667
O	-3.202358	2.476087	-0.112154
H	0.301362	0.121942	-1.261336
C	1.926026	0.878907	-0.133918
C	-0.598761	0.336544	0.637551
H	-0.351387	0.269554	1.697656
C	-1.704403	-0.611204	0.247814
C	-2.818553	0.076671	-0.043174
O	2.345238	1.078530	1.215036
H	2.062830	1.973342	1.454128
C	1.836118	2.257588	-0.810707
H	1.328692	2.179767	-1.776029
O	1.217451	3.223051	0.030122
H	0.273405	3.025222	0.097861
H	2.846285	2.626287	-0.984554
C	-4.156926	-0.371885	-0.510089
H	-4.470033	0.207370	-1.378901
H	-4.162075	-1.433684	-0.737717
H	-4.898403	-0.187304	0.270451

2C

C	1.465259	1.960007	0.224773
O	2.338479	2.766988	0.464360
C	-1.029693	1.427695	0.232059
C	-1.180401	1.633670	1.756650
H	-0.241906	1.504282	2.295276
H	-1.514814	2.654811	1.944860
H	-1.914133	0.964590	2.198318
C	0.075603	2.380501	-0.259267
H	0.112348	2.378983	-1.353308
H	-0.091725	3.409557	0.056823
C	-0.661157	-0.026203	-0.199354
C	2.404466	-1.626270	-0.213682
O	1.094795	-1.759791	0.158014
O	3.083833	-2.552185	-0.559148
H	-0.347469	0.020441	-1.244135
C	-1.983023	-0.834112	-0.258120
O	-1.783814	-1.829431	-1.281241
H	-2.637518	-2.209674	-1.512539
C	-2.425383	-1.568984	1.013670
C	0.570158	-0.485296	0.566694
H	0.361682	-0.574039	1.632253
C	1.692651	0.487940	0.286467
C	2.773272	-0.180819	-0.140051
H	-2.622631	-0.872561	1.826021
C	4.113514	0.291261	-0.574757
H	4.385396	-0.165683	-1.526392
H	4.150888	1.374379	-0.645954
H	4.866634	-0.028659	0.148978
C	-2.675483	1.133543	-1.797958
H	-1.859069	0.858778	-2.450051
H	-3.508506	1.592110	-2.310660
C	-2.410607	1.609140	-0.397370
H	-3.026827	2.411554	-0.014605
C	-3.009901	0.235157	-0.633910
H	-4.051068	0.034467	-0.416984
O	-1.472620	-2.494745	1.494508
H	-1.085118	-2.953075	0.738965
H	-3.377195	-2.070332	0.786127

2D

C	-1.504867	-1.988449	0.035993
O	-2.403833	-2.790406	0.169639
C	0.997186	-1.519537	0.155316
C	1.110198	-1.881379	1.654748
H	0.151087	-1.833843	2.172566
H	1.462797	-2.909883	1.744652
H	1.808086	-1.228356	2.170468
C	-0.114767	-2.383413	-0.466198
H	-0.128836	-2.257318	-1.554150
H	0.021976	-3.445662	-0.266301
C	0.673589	-0.013638	-0.112909
C	-2.363916	1.647639	-0.023056
O	-1.049986	1.707842	0.388550
O	-3.000718	2.626867	-0.286401
H	0.403817	0.075696	-1.168294
C	2.019061	0.746206	0.064604
C	-0.564207	0.375216	0.666202
H	-0.372193	0.338767	1.738663
C	-1.703850	-0.523649	0.258381
C	-2.761558	0.214256	-0.109484
C	-4.102359	-0.176762	-0.619089
H	-4.343153	0.381701	-1.523836
H	-4.159762	-1.245283	-0.804100
H	-4.865030	0.082505	0.118607
C	2.715854	-0.988970	-1.760832
H	1.926207	-0.634636	-2.407783
H	3.565366	-1.384076	-2.298663
C	2.393283	-1.651055	-0.449431
H	2.978772	-2.513608	-0.162721
C	3.023309	-0.272692	-0.465269
H	4.054696	-0.115782	-0.185532
O	2.318178	0.980587	1.441132
H	2.081362	1.903931	1.612648
C	2.090197	2.094566	-0.673155
H	3.135100	2.386895	-0.768529
H	1.667207	2.011141	-1.677114
O	1.468640	3.139217	0.069431
H	0.511338	3.004915	0.065682
